# The propionic acid and butyric acid in serum but not in feces are increased in patients with diarrhea-predominant irritable bowel syndrome

**DOI:** 10.1186/s12876-020-01212-3

**Published:** 2020-03-16

**Authors:** Zhenyi Tian, Xiaojun Zhuang, Mei Luo, Wei Yin, Lishou Xiong

**Affiliations:** 1 Department of Gastroenterology and Hepatology, the First Affiliated Hospital of Sun Yat-sen University, Guangzhou, 510080 China; 2 Department of Biochemistry, Zhongshan School of Medicine, Sun Yat-sen University, Guangzhou, 510080 China

**Keywords:** Gut microbiota, Diarrhea-predominant IBS, Short-chain fatty acids

## Abstract

**Background:**

Short-chain fatty acids (SCFAs) alteration have been reported in irritable bowel syndrome (IBS), but the results are conflicting. Our study aims to explore the alteration of SCFAs in patients with diarrhea-predominant IBS (IBS-D) and their potential role in the occurrence and development of IBS.

**Methods:**

We recruited patients with IBS-D defined by Rome IV criteria and age-and-gender matched healthy controls (HCs). A headspace solid-phase microextraction gas chromatography–mass spectrometric (HS-SPME-GC-MS) method was developed for the analysis of acetic, propionic and butyric acid in feces and serum.

**Results:**

Compared with HCs, the levels of the serum propionate (2.957 ± 0.157 vs 2.843 ± 0.098 mmol/L, *P* = 0.012) and butyrate (2.798 ± 0.126 vs 2.697 ± 0.077 mmol/L, *P* = 0.012) were significantly higher in IBS-D group. No significant differences were found among two groups with regard to the concentration of fecal acetate (4.953 ± 1.065 vs 4.774 ± 1.465 mg/g, *P* = 0.679), propionate (6.342 ± 1.005 vs 6.282 ± 1.077 mg/g, *P* = 0.868) and butyrate (2.984 ± 0.512 vs 3.071 ± 0.447 mg/g, *P* = 0.607).

**Conclusions:**

Metabolites of gut microbiota, the propionic and butyric acid, are increased in patients with IBS-D in serum but not in feces. It suggests that propionic and butyric acid might be associated with the occurrence and development of IBS.

## Background

Irritable Bowel Syndrome (IBS) is one of the most common functional bowel disorders characterised by recurrent or chronic abdominal pain accompanied by changes in bowel habits or associated with bowel movements [1]. It affects 7 to 21% of the population worldwide and 1 to 16% in China [2]. It is classified into Diarrhea-predominant IBS, Constipation- predominant IBS (IBS-C), Mixed type IBS (IBS-M), and Unspecified type IBS (IBS-U). The pathogenesis of IBS is complex that abnormalities of enteroendocrine and immune systems, genetic factors, infections and alterations of the intestinal microbiota could act a role in IBS [3]. A growing body of research suggests that alterations of gut microbiota might be closely associated with IBS. And gut microbiota might be involved in the pathogenesis of IBS by affecting brain-gut axis, activating immune reaction, disturbing gastrointestinal motility, altering mucosal permeability and inducing visceral hypersensitivity [4].

Short-chain fatty acids (SCFAs) are metabolites formed by gut microbiota from complex dietary carbohydrates. SCFAs, primarily acetate, propionate, and butyrate, play a pivotal role in maintaining homeostasis in humans. These three acids act on preserving gut barrier functions, and anti-inflammatory properties and immunomodulatory [5]. They are the most abundant (≥95%) in the total SCFAs [6]. It has been showed that SCFAs in human colon and stool are present in an approximate molar ratio of acetic: propionic: butyric acid of about 60: 20: 20 [7–10]. In the colon, about 95% of the produced SCFAs are rapidly absorbed by large intestinal mucosal cells while the remaining 5% are secreted in the feces [11]. A large part of absorption of SCFAs is used as a source of energy that provides about 10% of the daily caloric requirements in humans [12]. Propionate is only present at low concentration in the periphery because it is metabolized in the liver, leaving acetate as the most abundant SCFA in peripheral circulation [10]. Despite the low concentration in the peripheral circulation, propionate and butyrate could regulate cell signaling to affect peripheral organs as signal molecules [13]. The ability of SCFAs to modulate biological responses of the host depends on two major mechanisms. The first mechanism involves the direct inhibition of histone deacetylases (HDACs) to directly regulate gene expression to maintain modulators of immune homeostasis and maintenance of gut [13]. The second mechanism for SCFAs as signal molecules activate G-protein-coupled receptors (GPCRs) which mainly include GPR41, GPR43, and GPR109A [14].

SCFAs might play an important role in the occurrence and development of IBS, which is suggested by observations from animal and human studies. The gut microbiota of patients with IBS had change in diversity and richness [15, 16], which may impact the production of SCFAs in large intestinal. Hence, increasing studies have explored the association between IBS and SCFAs, and most of them measured the concentration of SCFAs in feces and few studies detect it in peripheral blood. For example, a study showed that fecal SCFAs in patients with IBS-D were decreased compared to HCs [17]. Another research demonstrated that there were no significant differences in the mean levels of fecal SCFAs between IBS but there were differences among subtypes [18]. These results are not entirely varying or even contradictory results. Indeed, someone illuminated that fecal SCFA could be used as a non-invasive, valid and reliable biomarker for the differentiation of healthy subjects from subjects with IBS [19]. Therefore, it is of great significance to measure SCFAs in human feces and clarify the relationship among the concentrations of acetic, propionic and butyric, and non-volatile short-chain fatty acids and IBS.

There are an increasing number of detection methods for SCFAs and quantification is getting more precise. Although SCFAs analytical methods have improved a lot in the past years, gas chromatography (GC) is still the most widely used quantification method of fecal SCFAs and its accuracy and rapidity cannot be surpassed by others [20]. Before the SCFAs analysis, the sample should be pretreated. Extraction and derivatization are two important pretreatment steps. Headspace solid-phase microextraction (HS-SPME) is an extraction technology that integrates extraction and derivatization. It can enhance selectivity and sensitivity, and increase the lifetime and the performance of the chromatographic system because it allows for a better clean-up of the matrix and reduces the presence of interfering compounds [21]. It is one of the most widely used ways to deal with the sample. Gas chromatography-mass spectrometry (GC-MS) was developed and validated for the analysis of SCFAs in fecal samples [22]. Therefore, we used HS-SPME-GC-MS to analyze the concentration of SCFAs in feces and serum in patients with IBS-D and HCs.

## Methods

### Study subjects

Twenty-one IBS-D patients who came in every Monday were enrolled from the outpatient clinic in the Department of Gastroenterology and Hepatology of the First Affiliated Hospital of Sun Yat-Sen University from September 2017 to November 2018, and 14 HCs were recruited by public advertising. All patients enrolled in this study fulfilled Rome IV diagnostic criteria for IBS and aged from 18 to 60. Patients were excluded if they had infectious gastroenteritis, organic gastrointestinal disease, previous abdominal surgery, lactose intolerance, metabolic diseases, human immunodeficiency virus infection and renal, cardiac or hepatic disease. Subjects taking any probiotics, prebiotics, antibiotics, or IBS prescription medications one month prior to baseline of our study were also excluded from our study. Age and gender matched HCs that they had no concomitant diseases, recurring GI symptoms, clinically significant abnormalities and medication taken.

### Chemicals

13C labeled acetic acid(= 99%, isotopic purity), 13C labeled propionic acid (= 99%, isotopic purity) and 13C labeled butyric acid (= 99%, isotopic purity) were purchased from Sigma-Aldrich (St. Louis, MO, USA). A 0.9 M H_2_SO4 solution was prepared by diluting H_2_SO4 (98% purity) (Guangzhou, China). Water was deionized by using a MQ-water (Millipore, Bedford, USA). Sodium chloride (Guangzhou, China).

### Fecal and serum samples

Twenty-one IBS-D patients and 14 HCs were asked not to take anything for at least 12 h before their stool samples and peripheral venous blood were collected. Samples could be obtained in the morning. Since SCFAs are volatile and feces contain high concentrations of microbes. In order to keep the biological material in appropriate conditions after its collection, samples were stored at − 80 °C until to be analyzed. The fecal sample was immediately homogenized, and then stored. Blood was centrifuged (4000 rpm, 15 min) and the serum was collected and stored at − 80 °C.

### Sample preparation

Ten stool samples with a mass of 0.5 g were separately added with 5 mL of 125 mg L^− 1^ three 13C labeled acids, with 2 g of sodium chloride (NaCl) and with 100 μL of a 0.9 M H_2_SO4 solution (pH = 2~3). Then the sample would be swirled for 5 min to homogenate. Finally, these vials were hermetically closed and submitted to test center in the south campus of Sun Yat-Sen University to extract. Follow these steps, the additional concentration of the three labeled acids were varied several times until it was approximately equal to the target acid in the samples. The same procedure is used for pretreatment of serum samples except for deproteinization with methanol. All samples were sent to the south test center of Sun Yat-Sen University for testing.

### Headspace solid-phase microextraction

Carboxen/polydimethylsiloxane (CAR/PDMS) 75 μm was applied to extract. The CAR/PDMS fibre gave best recoveries for the most volatile analytes like acetic and propionic acid [23]. HS-SPME conditions were as follows: extraction temperature 60 °C, extraction time 24 min and salt addition.

### Gas chromatography–mass spectrometry

The carrier gas was helium (pressure 115 kPa; flow 1.3 ml min^− 1^). Chromatographic separation was performed on a Supelcowax 10 fused-silica bonded-phase capillary column (30 m × 0.25 mm; film thickness = 0.25 μm; Supelco). GC oven temperature program was from 100 °C to 120 °C at 5 °C min^− 1^, then from 120°Cto 150°Cat 2 °C min-1, at last, from 150°Cto 240 °C at 30 °C min^− 1^ and the temperature should be1 min. The injector temperature was 250 °C; the interface and the source temperatures were 280 °C and 200°Crespectively. Electron impact mass spectra were recorded at 70 eV ionisation energy (scan time, 0.2 s; electron multiplier voltage, 700 V) scanning the mass spectrometer from 15 to 550 amu. Recorded mass spectra were compared with those stored in the National Institute of Standards and Technology (NIST) US Government library. Quantitative analysis was performed by measuring total ion current chromatographic peak areas. Firstly, the Thermo Xcalibur Roadmap software (Thermo Electron Corporation) was used to integrate the peaking areas of labeled acids and target acids in the tested sample. Then, the single point method was used for quantitative analysis of SCFAs, and concentration of target acids were calculated in the sample as follows: A_m_ = A_i_ * B_m_ /B_i_. where Am is the measured concentration of target acid in the sample and A_i_ is the peak area of the target acid. B_m_ and B_i_ represent measured concentration of 13C labeled acids and peak areas of 13C labeled acids in the sample solution injection volume. The amount of acetic, propionic and butyric acid in each sample was calculated by the above formula.

### Evaluation of method performance

**Fig. 1:**
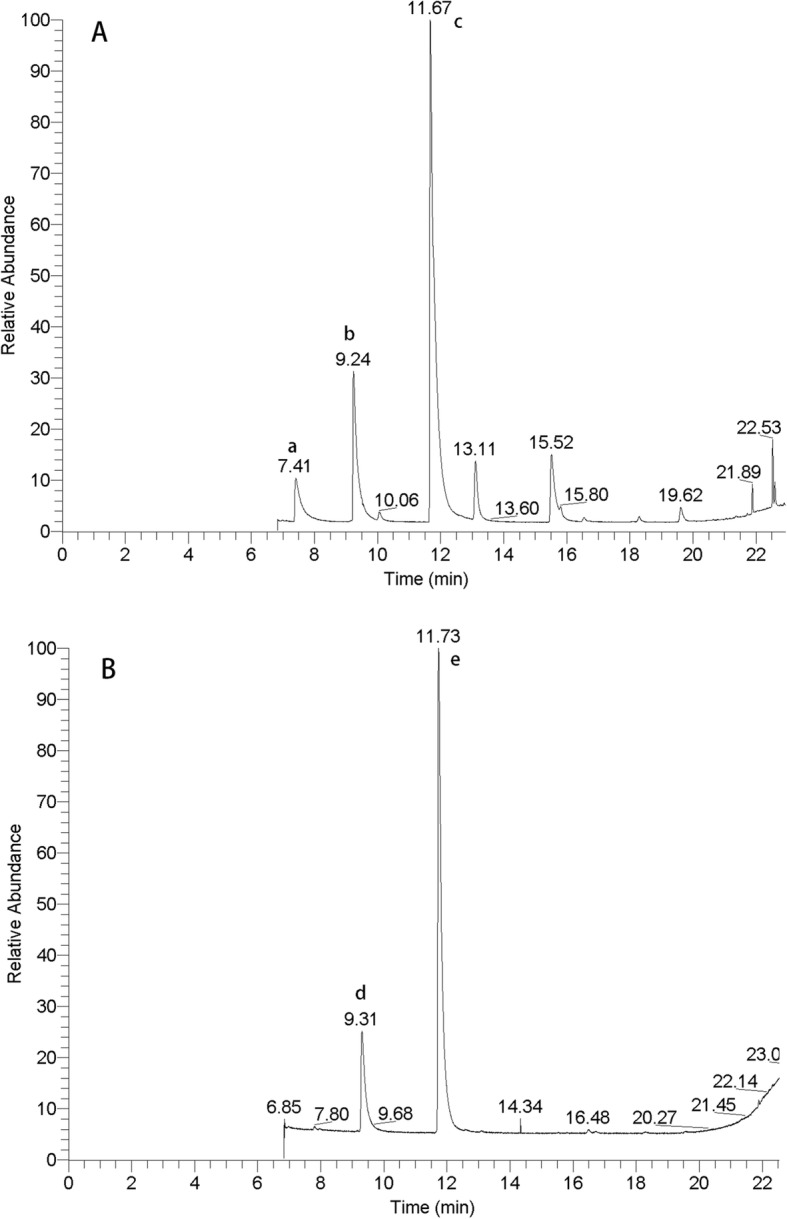
Gas chromatograms of SCFAs in feces A. Peak identification: a, acetic acid; b, propionic acid; c, butyric acid. The chromatograms of SCFAs in serum B. Peak identification: d, propionic acid; e, butyric acid

The chromatograms of SCFAs in feces and serum extracted are shown in Fig. 1. As can be seen, the peaks of SCFAs were very well separated. Chromatograms of extracted feces and serum reflected the high enrichment and high selectivity for the SCFAs.

As shown in Fig. 1. The names of several acids with peaks in the chromatogram are determined in the mass spectrogram by the properties of the proton of the acid. The peak at about 7 min was identified as acetate, while the peak at about 9 min was identified as propionate and the peak of butyric acid at approximately 11 min. The acetic acid in serum was undetected, it could not be detected even after doubling serum concentration.

The repeatability and reproducibility of the method were expressed as the relative standard deviation (RSD) of peak areas of 13C labeled acid. And the precision was evaluated with RSD (%). The acceptable precision was < 10%(RSD). The repeatability and reproducibility of the system before each injection were obtained an RSD 2.36% ~ 6.29%. Then, according to the difference of the number of protons in the target SCFAs and the labeled SCFAs, the chromatogram of the target SCFAs and the labeled SCFAs were separated and calculated.

### Statistical analysis

Statistical analysis was performed using the dedicated statistical software SPSS version 20.0 and Graph Prism version 7.0 (GraphPad software, Inc., La Jolla, CA, United States). The Student T-test was used for the comparisons between the groups. *P* values of < 0.05 were considered statistically significant.

## Results

### Characteristics of the subjects

Twenty-one patients with IBS-D (aged 19–54 years; mean 31.64 ± 8.85 years; BMI 20.39 ± 2.35 kg/m^2^; 8 females, 13 males) as well as 14 HCs (aged 20–40 years; mean 27 ± 4.38 years; BMI 20.32 ± 1.36 Kg/m^2^; 6 females, 8 males) participated in the study. There were no significant differences in gender, BMI or age between IBS-D patients and healthy controls. All patients and HCs completed the detection of SCFAs in feces and serum.

### Fecal SCFAs in patients with IBS-D

The peake areas of acids in the tested sample were integrated by the Thermo Xcalibur Roadmap software, and the concentration of three acids in feces were calculated. The statistical results are presented in Table 1.

**Table 1:** Faecal SCFAs in patients with IBS-D and HCs with comparisons between the groups

SCFAs (mg/g)	IBS-D (*n* = 21)	HC(*n* = 14)	*t value*	*P value*
Acetate	4.95 ± 1.07	4.77 ± 1.47	−0.42	0.68
Propionate	6.34 ± 1.01	6.28 ± 1.08	−0.17	0.87
Butyrate	2.98 ± 0.51	3.07 ± 0.45	0.55	0.61
Propionate –Butyrate	3.36 ± 1.10	3.21 ± 1.05	−0.40	0.69
Propionate/Butyrate	2.18 ± 0.50	2.08 ± 0.42	−0.67	0.51

T-test was adopted in group measurement data. SCFAS, short-chain fatty acids; IBS-D, diarrhea-predominant IBS; HC, healthy control

No significant differences were found between two groups with regard to the concentration of fecal acetic, propionic and butyric acid. Furthermore, the propionic/butyric ratio and the differences between propionic acid-butyric acid were calculated and showed no statistically significant differences between the groups.

### SCFAs in serum in patients with IBS-D

The concentration of SCFAs in serum was calculated according to the peak area of acid, and the statistical results are presented in Table 2.

**Table 2:** Propionate and butyrate in serum in patients with IBS-D and HCs

SCFAs in serum (mmol/L)	IBS-D (*n* = 21)	HCs(*n* = 14)	*t value*	*P value*
propionate	2.96 ± 0.16	2.843 ± 0.10	−2.65	0.01
butyrate	2.80 ± 0.13	2.70 ± 0.08	−2.67	0.01
Propionate –Butyrate	0.16 ± 0.23	0.15 ± 0.10	−0.21	0.84
Propionate/Butyrate	1.06 ± 0.08	1.05 ± 0.04	−0.22	0.83

T-test was adopted in group measurement data. SCFAS, short-chain fatty acids; IBS-D, diarrhea-predominant IBS; HC, healthy control

As compared to the control group, the levels of the serum propionic and butyric acid were significantly higher in IBS-D group. But no significant differences were found between two groups about the propionic/butyric ratio and Prop-But. The acetic acid in serum was undetected.

## Discussion

Gut microbiota might play an important role in IBS-D, and the relationship between the metabolites of gut microbiota and IBS-D has received increasing attention. As products of gut microbiota, SCFAs may reflect the status of the microbiota. As early in 1987, Mortensen et al. found that fecal SCFAs increase in patients with IBS-D [24]. But there are some inconsistent reports. Other studies demonstrated that fecal SCFAs could be reliable biomarkers for the differentiation of healthy subjects from subjects with IBS [19]. The concentrations of SCFAs in serum and feces were measured in this study. It was acted as surrogate markers for intraluminal intestinal fermentation and used these factors to assess the magnitude of intestinal bacterial fermentation in a well characterized cohort of patients with IBS-D and HCs and to investigate the role of SCFAs in IBS-D.

HS-SPME coupled with GC-MS that were applied to analyze the concentration of SCFAs of patients with IBS-D and HCs in feces and serum. HS-SPME is mainly used for the extraction of volatile substances such as SCFAs. The technique is a relatively inexpensive, fast and easily automated sample preparation one [21, 25]. It depends on the use of a short fused-silica fibre clean-up and concentration in a single step and reduces the presence of interfering compounds and the loss of samples. We used it thus enhancing selectivity and sensitivity, making it possible to better discriminate volatile acid [23]. The CAR/PDMS fibre was selected for extraction because it gave high recoveries for the most volatile analytes like acetic, propionic and butyric [23]. NaCl was used to improve the extraction efficiency [26]. GC-MS is often used for detecting SCFAs in biological samples, and its reliability has been verified [22, 23, 27–29]. The matrix of feces and serum is complex, and the standard curve is difficult to measure and easy to be affected. Therefore, we adopted the single-point method for quantitative analysis and 13C labeled acetic, propionic and butyric acid were internal standard. The repeatability and reproducibility of the method were verified before each measurement and RSD was always lower than 10%.

Our study indicated that there are no significant differences between two groups about the level of SCFAs in feces. Ringel-Kulka et al. [18] found that there were no significant differences in the mean levels of fecal SCFAs between IBS and HC and the levels of fecal SCFAs did not correlate with IBS symptom severity, and they thought that fecal SCFAs might not be a sensitive marker to estimate intraluminal bacterial fermentation. However, studies in fecal SCFAs have generated conflicting findings. For example, a study conducted in patients with IBS-D showed that fecal SCFAs were increased compared to HCs [24]. There was a study showed that the fecal SCFA profile of patients with IBS-D is characterized by lower concentrations of total SCFA, acetate, and propionate and a higher concentration and percentage of butyrate [17]. Germana et al. [30] found that acetate and propionate were significantly higher in IBS-D compared to HCs; in addition, the levels of acetate, butyrate, propionate and valerate were significantly higher in IBS-D than in IBS-C. But Farup et al. [19] suggested that there was a non-significant trend toward a higher concentration of propionic acid in IBS-D group, whereas no significant differences were found between two groups about acetic and butyric acid in feces; they also have calculated the propionic/butyric ratio and Prop-But and showed highly statistically significant differences between the groups, while Prop-But (mmol/L) was the best one for the discrimination between IBS and HCs, and they implied that SCFAs showed very satisfactory diagnostic properties for the diagnosis of IBS. However, we found no significant differences with respect to the fecal propionic/butyric ratio and propionic acid-butyric acid in patients with IBS-D and HCs. Others also agreed that fecal SCFAs could be used as a biomarker for the discrimination of IBS from HCs [31]. A recent systematic review and meta-analysis demonstrated that fecal butyrate was increased in IBS-D patients in comparison to HCs, and fecal propionate and butyrate could be used as biomarkers for IBS diagnosis [32]. However, someone suggested that fecal SCFAs concentrations do not reflect their concentration and production rate in the colon because most SCFAs are absorbed by the host and therefore fecal SCFAs provide little information about actual intestinal SCFAs yield [12]. Indeed, the concentration of fecal SCFAs is affected by many factors, such as colonic transit time and the structure of gut microbiota.

Jakobsdottir et al. [33] stated that fecal SCFAs do not necessarily represent colonic SCFAs levels and analysis in blood may in fact be a better alternative as the vast majority of SCFAs are absorbed from the colon. Meanwhile, Jakobsdottir et al. [34] demonstrated that there is a correlation between cecal levels of SCFA and portal and aortic blood levels of SCFA in rats. There have been few studies to explore SCFAs in serum in patients with IBS so far, which might be due to the shortage of appropriate analytical methods. We found that there are increases of propionic and butyric acid in serum of patients with IBS-D. Undseth et al. [35] found that fasting serum levels of SCFAs did not differ from patients with IBS and HCs, but they did not distinguish the IBS subtypes in their study.

We found that the concentration of acetate was undetected in serum in patients with IBS-D and HCs while fecal acetate was measured. SCFAs are the major metabolites of the microbial that dietary fibers and proteins and peptides, undigested in the intestine, are metabolized by the microbiota in the cecum and colon [13]. Acetate, propionate, and butyrate account for about 95% of the total, and 95% of the produced SCFAs in the cecum and large intestine are rapidly absorbed by the colonocytes but the remaining 5% are secreted in the feces [12]. More than 70% of the acetate that used as an energy source as well as a substrate and a cosubstrate for somethings synthesis is taken up by the liver while the remainder of it is metabolized by other tissues [36, 37]. Around 30% of propionate is taken up by the liver as a precursor for gluconeogenesis and butyrate as the major energy source for intestinal epithelial cells [12]. Patients with IBS-D and HCs were asked to fast for 12 h before serum and feces were collected in our study. Acetate in serum might be metabolized as energy that resulted in the concentration of acetic acid lower than the lowest detection limit, which might be the reason why the concentration of acetate in serum was undetected. Nevertheless, it needs some basal metabolomics studies to validated.

It becomes increasingly apparent that SCFAs play a prominent part in the prevention and treatment of some diseases, such as IBS, metabolic syndromes, colonitis, and certain types of cancer [38]. SCFAs could enhance the gut epithelial barrier and accelerate the repair of it. The gut epithelial barrier is composed of epithelial cells, antimicrobial products and a mucus layer. Barcelo et al. [39] confirmed that acetate and butyrate facilitate the release of mucin. Macia et al. [40] implied that SCFAs promote gut epithelial integrity through the inflammasome pathway. Although moderate SCFAs could stabilize intestinal permeability by directly regulating the distribution of tight junction proteins, high concentration of SCFAs is more likely to have the opposite effect [41, 42]. The increase of intestinal permeability is one of the causes of IBS-D [43]. SCFAs could involve in the pathogenesis of IBS through excessive activation of intestinal immunity. They are involved in regulation of intestinal immune and play a vital role in intestinal resistance to pathogenic bacteria by affecting the release of inflammatory cytokines, immune chemotaxis, and inhibiting the proliferation of immune effector cells [44]. It was reported that butyrate and propionate could induce the differentiation of T-regulatory cells and assist to restrain intestinal inflammation; they seem to be mediated via inhibition of histone deacetylation [45]. The gut serotonergic system plays a crucial role in modulating peripheral mechanisms implicated in IBS. Dunlop et al. [46] showed that patients with IBS-C postprandial 5-hydroxytryptamine (5-HT) were damaged release while they have higher peak levels. Tryptophan hydroxylase 1 (Tph1) is important for normal 5-HT production by gut mucosal EC cells. Reigstad et al. [47] found the result that gut microbiota accelerate colonic Tph1 expression and 5-HT production through stimulatory activities of SCFAs on EC cells, which indicated that SCFAs are key determinants of gut microbiota in maintaining enteric 5-HT production and homeostasis. Hence, it is reasonable to believe that SCFAs may also associate with the pathogenesis of IBS-D through the brain-gut microbiota axis. SCFAs also might relate to visceral hypersensitivity. It was reported that SCFAs would cause abdominal pain when it was injected into the ileum of healthy persons [48]. Matricon et al. [49] implied that butyrate is related to an increase in visceral hyperalgesia of rat in their study. A previous study showed that there is an association between organic acids produced by gut microbiota and IBS symptoms on the visceral sensation indicates that increased chemical incentive could be one of the origins or aggravation factors in IBS [50]. It is reported that SCFAs might involve in regulating intestinal motility but those findings are not consistent. Recently, Mazzawi et al. [51] explored the effects of fecal microbiota transplantation on gut microenvironment and analyzed bacterial fermentation products in patients with IBS-D, and they confirmed the associations that both normal the levels of SCFAs and gut microbiota may be beneficial to IBS between gut microbiota, SCFAs and IBS symptoms.

A limitation is that dietary habit could affect SCFAs production, however, we did not control the diet of them. The other one, acetate is not measured in serum in two groups, and it lacks further experimental of metabolomics to analyze. Besides, we failed to further study the relationship between diarrhea and SCFA in our study and we acknowledged that this is a limitation of our study.

## Conclusion

We explored variations of SCFAs in feces and serum in patients with IBS-D. There are increases of propionic acid and butyric acid in serum but not in feces in patients with IBS-D in this study. The gut microbiota might be associated with the occurrence and development of IBS through propionic and butyric acid. SCFAs are associated with IBS would be a useful focus for future studies. Due to the limitations of the present study, further investigation into SCFAs in IBS patients, and a probe of gut microbiota–IBS is warranted.

## Data Availability

All data and materials are not available in this study, and are available from the corresponding author on reasonable requests.

## Abbreviations

HCs: Healthy controls
HS-SPME-GC-MS: Solid-phase microextraction gas chromatography–mass spectrometric
IBS: Irritable bowel syndrome
IBS-D: patients with diarrhea-predominant IBS
SCFAs: Short-chain fatty acids
